# An Objective Approach to Select Climate Scenarios when Projecting Species Distribution under Climate Change

**DOI:** 10.1371/journal.pone.0152495

**Published:** 2016-03-25

**Authors:** Nicolas Casajus, Catherine Périé, Travis Logan, Marie-Claude Lambert, Sylvie de Blois, Dominique Berteaux

**Affiliations:** 1 Canada Research Chair on Northern Biodiversity and Centre d’Études Nordiques, Université du Québec à Rimouski, 300 allée des Ursulines, Rimouski, QC, G5L 3A1, Canada; 2 Direction de la recherche forestière, Ministère des Forêts, de la Faune et des Parcs, Québec, QC, G1P 3W8, Canada; 3 Ouranos Consortium on Regional Climatology and Adaptation to Climate Change, Montréal, QC, H3A 1B9, Canada; 4 Department of Plant Science and McGill School of Environment, Macdonald Campus, McGill University, 21111 Lakeshore Road, Ste-Anne-de-Bellevue, QC, H9X 3V9, Canada; University of Sydney, AUSTRALIA

## Abstract

An impressive number of new climate change scenarios have recently become available to assess the ecological impacts of climate change. Among these impacts, shifts in species range analyzed with species distribution models are the most widely studied. Whereas it is widely recognized that the uncertainty in future climatic conditions must be taken into account in impact studies, many assessments of species range shifts still rely on just a few climate change scenarios, often selected arbitrarily. We describe a method to select objectively a subset of climate change scenarios among a large ensemble of available ones. Our k-means clustering approach reduces the number of climate change scenarios needed to project species distributions, while retaining the coverage of uncertainty in future climate conditions. We first show, for three biologically-relevant climatic variables, that a reduced number of six climate change scenarios generates average climatic conditions very close to those obtained from a set of 27 scenarios available before reduction. A case study on potential gains and losses of habitat by three northeastern American tree species shows that potential future species distributions projected from the selected six climate change scenarios are very similar to those obtained from the full set of 27, although with some spatial discrepancies at the edges of species distributions. In contrast, projections based on just a few climate models vary strongly according to the initial choice of climate models. We give clear guidance on how to reduce the number of climate change scenarios while retaining the central tendencies and coverage of uncertainty in future climatic conditions. This should be particularly useful during future climate change impact studies as more than twice as many climate models were reported in the fifth assessment report of IPCC compared to the previous one.

## Introduction

All ecological projections of the impacts of climate change ultimately rely on models simulating climate change based on scenarios of anthropogenic forcing (Table 1). For its fifth assessment report (AR5), the Intergovernmental Panel on Climate Change (IPCC) has selected new climate model simulations carried out under the framework of the Coupled Model Intercomparison Project Phase 5 (CMIP5), as well as new forcing scenarios, the Representative Concentration Pathways (RCPs). This has resulted in an impressive number of new climate change scenarios (Table 1) now available to conduct climate change impact studies. For example, 138 global mean temperature projections for 2050 relative to 1986–2005 are presented in AR5 [1]. Each was obtained from one of four RCPs combined to many (from 25 to 42) coupled atmosphere-ocean general circulation models (AOGCMs).

**Table 1:** Glossary of important terms used in this paper.

Term	Definition
Climate model	Complex mathematical representation of the Earth ’s climate system coupling many physical processes (i.e. atmosphere flux, ocean circulation, land surface and sea ice dynamics, snow cover, and permafrost) [2].
Forcing scenario	Hypothesis of the modification of the balance of incoming and outgoing energy in the Earth-atmosphere system. The new generation of forcing scenarios, the Representative Concentration Pathways (RCPs), provides potential greenhouse gas concentration trajectories for the future whereas the Emissions Scenarios (SRES) hypothetize the greenhouse gas emissions in the atmosphere.
Initial conditions	In climate models (chaotic systems), initial conditions refer to starting values of climate variables at a given place and time. Small changes in these starting values could lead to different paths of the climatic system.
Climate change scenario	Potential future climate projected by a climate model under a specific forcing scenario and initial conditions. In general climate change scenarios are available for 30-year periods.
Ensemble forecasting	Large number of copies of a system, each of which represents a possible state of this system. In climate change science, ensemble forecasting consists in using climate change scenarios obtained from different climate models run under different forcing scenarios and different initial conditions.

Climate models (Table 1) are complex mathematical representations of the Earth’s climate system as they couple many physical processes such as atmosphere flux, ocean circulation, land surface and sea ice dynamics, snow cover, and permafrost [2]. They differ from each other [2, 3], notably in the parameters and functions used to describe the physical processes of the ocean and atmosphere circulations. Forcing scenarios also differ from each other as they provide alternative hypotheses about the development of human society, through different demographic, social, political, technological, and environmental assumptions [4]. To address uncertainty in projected changes, the IPCC [5] thus recommends using a large ensemble of climate change scenarios (Table 1) produced from various combinations of AOGCMs and forcing scenarios. Importantly, all climate change scenarios provided by IPCC should be considered plausible and illustrative, and do not have probabilities attached to them [1].

**Fig 1:**
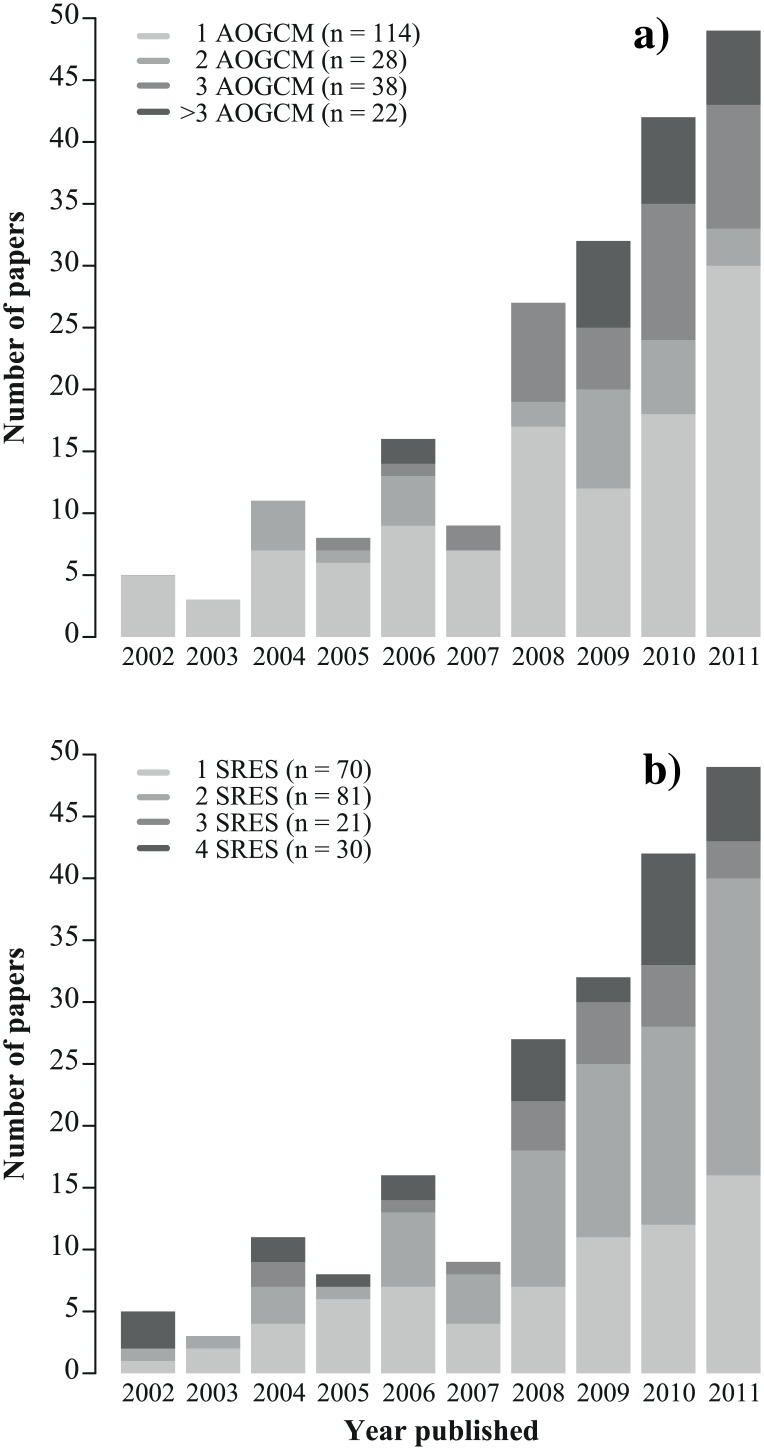
Use of climate models and emissions scenarios in papers reporting species distribution models. Histograms show the number of publications per year (2002–2011) using 1, 2, 3 or > 3 coupled atmosphere-ocean general circulation models (AOGCMs) (a) and 1, 2, 3 or 4 emissions scenarios from the special report of emissions scenarios (SRES) (b) to project species distribution under climate change. Data come from a literature search in ISI Web of Science performed on June 5, 2013 and using the following search settings: model* AND species distribution OR ecological niche OR habitat suitability OR bioclimatic envelope OR environmental niche OR habitat distribution OR niche-based AND climat* change OR global warm*.

In ecology, climate change scenarios are commonly used with species distribution models (SDMs) to assess shifts in species range induced by climate change [6, 7]. SDMs correlate the observed distribution of a species to a set of environmental predictors, including climate, and use this relationship to project the potential distribution into the future [8–10]. Despite their limitations [11, 12], SDMs provide a useful first approximation of the direction and magnitude of potential impacts of climate change on species range. Like for AOGCMs, many SDMs are available to model the distribution of a given species, due to the various statistical models and calibration and evaluation datasets available during SDM construction. It is thus standard practice to use, in any single study, several SDM outputs in an ensemble framework [13]. However, it can become prohibitively time consuming to assess the impacts of climate change on many species, using simultaneously many climate change scenarios and many SDMs. As a result, researchers typically project species distributions under only one or a few climate change scenarios. From 2002 to 2011, 55% of the studies that have projected species distribution under climate change used a single AOGCM (Fig 1a) and 78% of them used only one or two forcing scenarios (Fig 1b). Moreover, researchers often select climate change scenarios arbitrarily or based on logistic constraints, and provide little or no justification about their choice. Yet different modelling frameworks can lead to different projections of species distribution [14–16], and possibly to conflicting interpretations [17].

In this context, a critical question is which and how many climate change scenarios are required to carry out impact analyses that cover the range of possible climate futures. Surprisingly, there is no publication aimed at presenting and testing an objective method to select an appropriate subset of climate change scenarios among the wide range of possibilities [18] (but see [19]). Given the importance of both taking into account the wide range of equally probable climatic futures and avoiding computationally prohibitive study designs, developing an objective method that reduces the number of climate change scenarios needed to project species distributions while retaining the coverage of uncertainty in future climatic conditions would constitute an important methodological progress. Here we describe and test such a method.

## Materials and Methods

### Study design

We first describe a k-means clustering approach allowing the objective selection of a subset of climate change scenarios from a large group of 27 derived from nine AOGCMs coupled with three forcing scenarios. We analyze the size and composition of the clusters obtained from this approach and compare, for three biologically-relevant climatic variables, the distribution of values obtained from the subset to that obtained from all 27 climate change scenarios. Secondly, we test the added value of the k-means clustering approach when projecting changes in species distribution, through a case study involving potential gains and losses of habitat by three northeastern American tree species. To do so, we compare future species habitat distributions projected from the subset of climate change scenarios (our proposed method) with those obtained from the full set of 27 climate change scenarios, as well as with those resulting from an arbitrary selection of just a few AOGCMs (the common practice).

Due to data availability when conducting this study, we worked with the forcing scenarios of the Special Report on Emissions Scenarios (SRES) [20] and the climate model simulations of the third phase of the Coupled Model Intercomparison Project (CMIP3), both used in AR4, rather than with the RCPs and climate model simulations of the CMIP5, used in AR5. However, our suggested approach remains entirely valid for the climate change scenarios used in AR5, or for those to be used in future assessment reports of the IPCC.

### Study area

This study is part of a larger project [21, 22] exploring the impacts of climate change on Quebec biodiversity. Although focused on Quebec, the study area covers all the U.S. states east of the 100^th^ meridian (excluding Florida) because many species found in Quebec are also present more to the South, and their niche cannot be modelled adequately without distribution data from the central parts of their range. We gridded our study area (9,613 cells of 20 km x 20 km) using the grid developed by Prasad *et al.* [23], and extending it to Quebec.

### Species data

We modelled current and future habitat distributions of the American Beech (*Fagus grandifolia*), the Pitch Pine (*Pinus rigida*), and the Blackjack Oak (*Quercus marilandica*). We chose these three species because they represented different patterns of spatial extent and range boundaries. For the U.S., we obtained presence/absence data online from the USDA tree Atlas website [24] whereas we obtained Quebec data from an extensive database of more than 95,000 forest plots of the third decennial inventory of the Ministry of Forests, Wildlife and Parks. We aggregated the plot level presence/absence data from Quebec at the 20 km x 20 km cell resolution.

### Climate data

We related species distribution to climate using three climatic variables that influence plant survival and growth: mean annual temperature (°C), total annual precipitation (mm), and useful precipitation (ratio of summer precipitation to total annual precipitation). These three variables describe the main climatic gradients while reducing the multicollinearity which biases parameter estimation in SDMs. We derived these three climatic variables for the reference period (1961–1990) from climatic surfaces available at the U.S. Forest Service, Rocky Mountain Research station website (http://forest.moscowfsl.wsu.edu/climate). Further details on these interpolated weather station data are available in Rehfeldt [25]. We downloaded these data with a resolution of 0.0083 decimal degrees (≈ 1 km), and subsequently averaged for each 20 km x 20 km grid cell.

We produced future climate scenarios using output from nine AOGCMs (Table 2) available through CMIP3 [26]. These nine AOGCMs were those, among a larger ensemble, that were available for our purposes because they simulated climate in our study area for 1961–1990 atmospheric conditions as well as for anticipated conditions under three SRES scenarios (A2 family, A1B, and B1, [20]. In total, 27 future climate scenarios were thus available for our purposes (Table 2). For each we obtained temperature and precipitation data for 2071–2100 using the “change field” method [27] (see also S1 Text).

**Table 2:** Description of the climate change scenarios used in this study.

Center	AOGCM	SRES	ΔTavg (°C)	ΔPrec (%)	ΔPrat (%)
CCCMA	CGCM3.1 (T47)	A2	+5.0	**+18.2**	−4.2
		A1B	+3.9	+15.3	−3.6
		B1	+2.8	+7.7	−3.2
CNRM	CM3	A2	+4.5	+9.4	−4.0
		A1B	+3.5	+8.4	−3.1
		B1	+2.2	+5.4	−2.5
CSIRO	MK3.0	A2	+3.9	+11.8	−1.6
		A1B	+2.8	+6.7	−2.8
		B1	**+1.8**	+4.8	−1.5
CSIRO	MK3.5	A2	+4.7	+11.0	−2.7
		A1B	+3.9	+15.4	−0.7
		B1	+2.9	+7.3	−2.5
GFDL	CM2.0	A2	+5.2	+3.9	−7.4
		A1B	+4.3	+9.6	−3.4
		B1	+2.8	+8.1	−2.0
IPSL	CM4	A2	**+6.9**	+2.8	−2.4
		A1B	+5.8	+5.4	−2.8
		B1	+4.2	+1.6	−2.2
CCSR	MIROC3.2 (Medres)	A2	+6.8	**−0.8**	**−9.3**
		A1B	+5.8	+3.1	−7.1
		B1	+4.0	+5.7	−5.2
MIUB	ECHO-G	A2	+5.0	+12.6	−0.1
		A1B	+4.8	+11.4	−0.2
		B1	+3.4	+5.9	**+0.3**
MRI	CGCM2.3.2	A2	+3.7	+13.7	−1.1
		A1B	+3.3	+11.0	−1.3
		B1	+2.5	+8.9	+0.2
Average ± SD			+4.1 ± 1.3	+8.3 ± 4.5	−2.8 ± 2.3

Each scenario combines an atmosphere-ocean general circulation models (AOGCM) from a given research center to an emissions scenario from the special report of emissions scenarios (SRES). ΔTavg: projected change in average annual temperature between 1961–1990 and 2071–2100 for the study area; ΔPrec: projected change in total annual precipitation; ΔPrat: projected change in useful precipitation (ratio of summer precipitation to total annual precipitation). Minimum and maximum values for each variable are in bold character.

### Description of the approach used to select climate change scenarios

We used the k-means clustering approach [28] to select climate change scenarios. This method iteratively partitions *n* objects, described by *p* variables, into *k* clusters in which each object belongs to the cluster with the nearest cluster centroid. The choice of initial seeds (i.e. started values of the cluster centers) is important and we followed Peterson *et al.* [29] who recommend the use of a hierarchical clustering method to define initial seeds for the k-means algorithm [30].

First, we built a climate distance matrix using Euclidean distances between the 27 climate change scenarios (Table 2) described by the three standardized climatic variables (Step 1). Standardization is necessary in order to avoid differences in units having a weighting effect on the clustering algorithm. Then, we applied hierarchical clustering on this distance matrix using the Ward’s minimum variance method [31] as the agglomeration criterion (Step 2). From this first grouping, we isolated *k* clusters and calculated their centroids (Step 3). Next, we performed a k-means clustering where initial seeds corresponded to the cluster centroids calculated from the hierarchical clustering (Step 4). The iterative process during which cluster centers are recalculated was performed 999 times in order to find the optimum partitioning with *k* clusters. Finally we calculated the ratio of the between-group sums of squares to the total sums of squares (herein referred as the Rsq statistic; Step 5), which quantifies the amount of variability captured by the clustering.

In order to determine an appropriate value of *k*, we repeated steps 3 to 5 by varying *k* from 1 to 27. The number of clusters to be used can be determined by evaluating the degree of group partitioning using an Rsq profile plot describing the Rsq statistic as a function of the number of clusters (S2 Text). We determined the optimal number of clusters under a logic based on a trade-off between costs (number of clusters) and benefits (explained variance), and we identified the number of clusters from which the net benefit decreases (see details in S2 Text). Finally, for each cluster, we selected the climate change scenario that was nearest to the cluster center for use in subsequent SDM projections.

### Species distribution modelling

We modelled the distribution of *Fagus grandifolia*, *Pinus rigida* and *Quercus marilandica* using seven statistical algorithms implemented in the BIOMOD package [32] developed for the R statistical software [33]. These seven statistical models included two regression techniques (generalized additive models and generalized linear models), two classification approaches (mixture discriminant analysis and classification tree analysis), and three machine learning methods (artificial neural networks, generalized boosted models and random forest).

We randomly split the initial dataset in two datasets to evaluate predictive performance of models on pseudo-independent data [34]. The first dataset was a calibration dataset containing 70% of the data, while the second was an evaluation dataset containing the remaining 30%. We repeated this split-sample procedure 20 times. For each species, we thus calibrated 140 SDMs (20 datasets x 7 statistical models). We evaluated predictive performance of each of these models using the area under the curve (AUC) of the receiver-operating characteristic (ROC) plot [35].

From these calibrated models, we simulated the potential distribution of the three species habitat for the reference period (1961–1990) and obtained 140 probabilities of occurrence by grid cell for each species. We simulated future habitat distributions by projecting models under each of the 27 climate change scenarios for the period 2071–2100, and thus produced 3,780 future potential probabilities of occurrence (140 SDMs x 9 AOGCMs x 3 SRES emissions scenarios) per grid cell for each species.

### Aggregating projections of species distributions for the reference period

We summarized, for each species, the 140 distribution projections for the reference period using a consensus technique [13], aggregating probabilities of occurrence using the weighted average approach [36]. We weighted probabilities of occurrence by the AUC of their corresponding models and averaged them to produce a single probability of occurrence per grid cell for the period 1961–1990. Then, we transformed these consensual probability values into presence/absence data by using the sensitivity-specificity sum maximization approach [37]. Although some information is lost when consensual probabilities are transformed into presence/absence data, this was needed to calculate percentages of grid cells projected to be gained or lost by species, a standard practice in climate change biology [38–40].

### Aggregating projections of species distributions for 2071–2100

After projecting species habitat distribution under climate change scenarios selected by the k-means approach, we summarized the range of projections using the weighted average approach. Because the size of clusters was heterogeneous, we also weighted the future probabilities of occurrence by the number of scenarios in each cluster, to avoid an over-representation of climate change scenarios from small clusters. Future projections of species habitat distributions obtained under the climate change scenarios selected by the k-means algorithm were thus double-weighted, according to Eq (1), where ${\bar{x}}^{*}$ is the weighted average of probabilities of future occurrence for a given pixel, *x*_*ij*_ is the probability of future occurrence obtained under the statistical model *i* coupled with the climate change scenario *j* for the same pixel, *AUC*_*i*_ is the AUC of the statistical model *i*, *n* is the total number of calibrated statistical models (here, n = 140), *nk*_*j*_ is the number of climate change scenarios in the cluster *j*, and *k* is the total number of selected clusters.

$${\bar{x}}^{*}=\sum_{i=1}^{n}\sum_{j=1}^{k}x_{ij}\frac{AUC_{i}}{\sum_{m=1}^{n}AUC_{m}}\frac{nk_{j}}{\sum_{p=1}^{k}nk_{p}}$$ (1)

As one of our objectives was to compare outcomes from the k-means algorithm with those obtained from the full set of 27 climate change scenarios, we also weighted averaged future species habitat distributions projected under the 27 climate change scenarios. These probabilities of occurrence were simple-weighted by the AUC of their corresponding models.

We transformed consensual future probabilities of occurrence in a presence/absence form, as had been done for consensual probabilities of occurrence for the reference period by using the transformation thresholds calculated for the reference period. This allowed us calculating the percentage of grid cells projected to be gained (i.e. the number of cells where the species was absent during the reference period but will potentially be present in the future if it colonizes newly available climatic habitat, divided by the total number of cells where it was present during the reference period) or lost (i.e. the number of cells where the species was present during the reference period but will potentially be absent in the future, divided by the total number of cells where it was present during the reference period) by a given species. We assumed for this exercise an unlimited dispersal scenario.

### Impact of an arbitrary selection of AOGCMs on projected species distribution

Since it is common practice during impact studies to select just a few AOGCMs arbitrarily, we performed a sensitivity analysis to assess the impact of an arbitrary selection of AOGCMs on future projected species distribution. We aggregated by the weighted average approach (using the AUC as the weight) the projected future species distributions obtained from *q* AOGCMs, with *q* varying from 1 to 9 (the total number of available AOGCMs for this study). For each AOGCM, we used the three available SRES emissions scenarios. For each value of *q*, we selected all possible combinations of AOGCMs. For example, if two AOGCMs had to be selected, 36 combinations of two AOGCMs were possible. In this case, 36 consensual projections were performed, each resulting in a weighted average of 840 future projections (140 SDMs x 2 AOGCMs x 3 SRESs). For each combination of AOGCMs, we also computed the percentages of species gains and losses, assuming unlimited dispersal.

We did all the analyses using the R statistical software [33] and performed cartography using arcgis Desktop version 9.3.1 (ESRI, Redlands, CA, USA).

## Results

### Selection of climate change scenarios

**Fig 2:**
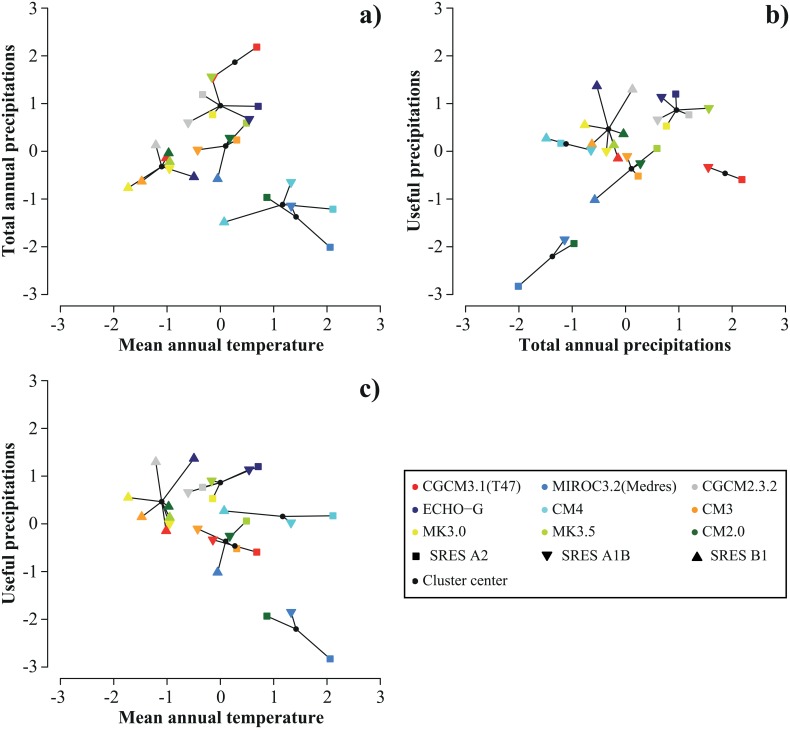
Clustering of climate change scenarios. Scatter plots show the clustering of climate change scenarios in two dimensions: standardized delta value (Δ) of total annual precipitation as a function of Δ mean annual temperature (a), Δ total annual precipitation as a function of Δ useful precipitation (b), Δ useful precipitation as a function of Δ mean annual temperature (c).

The k-means clustering led to six clusters summarizing 83% of the variance in the climate change scenarios (S2 Text). Cluster size varied from two to eight climate change scenarios and the composition of clusters did not reflect consistently AOGCMs or forcing scenarios (Fig 2). With the exception of CM4, all AOGCMs belonged to ≥ two clusters (Fig 2).

**Fig 3:**
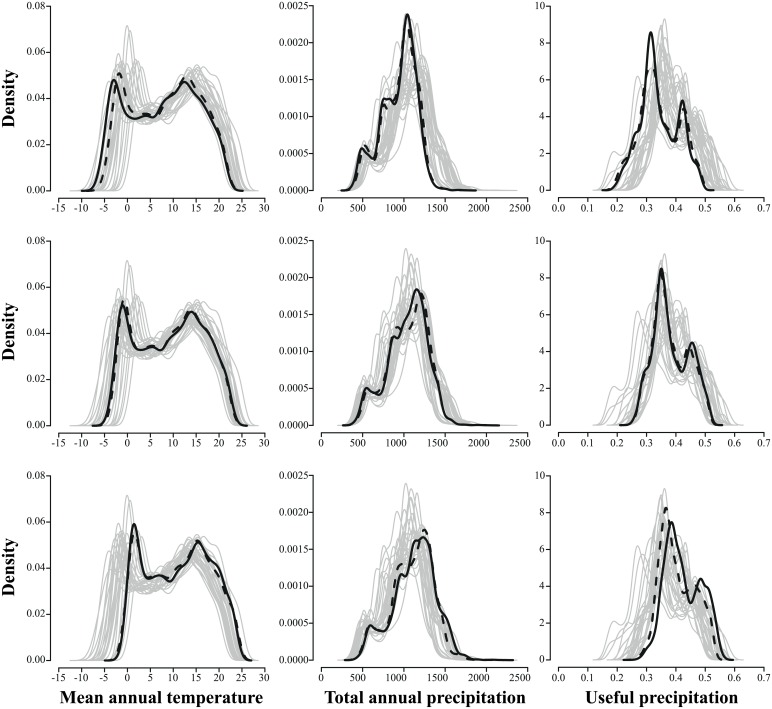
Projected climate for three biologically-relevant variables. Graphs show probability density functions of projected climate for mean annual temperature (first column), total annual precipitation (second column) and useful precipitation (third column). The 27 climate change scenarios are plotted as gray lines. The solid and dashed black lines represent the 10^th^ percentile values (top row), the average values (mid row), and the 90^th^ percentile values (bottom row) calculated on each cell across the 27 climate change scenarios (solid lines) or the six climate change scenarios selected by the k-means algorithm (dashed lines).

For the three analyzed climatic variables, both the range and the distribution of projected values were very similar between the average obtained from all the 27 climate change scenarios and the weighted average (using the number of climate change scenarios by clusters as the weight) obtained from the six climate change scenarios selected by the k-means algorithm (Fig 3, mid row). Both 10^th^ (Fig 3, top row) and 90^th^ percentiles (Fig 3, bottom row) show similar patterns in the distribution of the projected values of the three climatic variables.

### Performance of species distribution models

*Quercus marilandica* showed the lowest mean predictive performance (AUC = 0.86 ± 0.08 SD; see also S3 Text). However, according to the interpretation of the AUC values [41] models still remained accurate to project the potential future habitat distribution of this species. *Fagus grandifolia* and *Pinus rigida* showed good to excellent predictive performances (AUC = 0.89 ± 0.04 SD and 0.90 ± 0.09 SD, respectively; see also S3 Text).

### Impact of an arbitrary selection of AOGCMs

**Fig 4:**
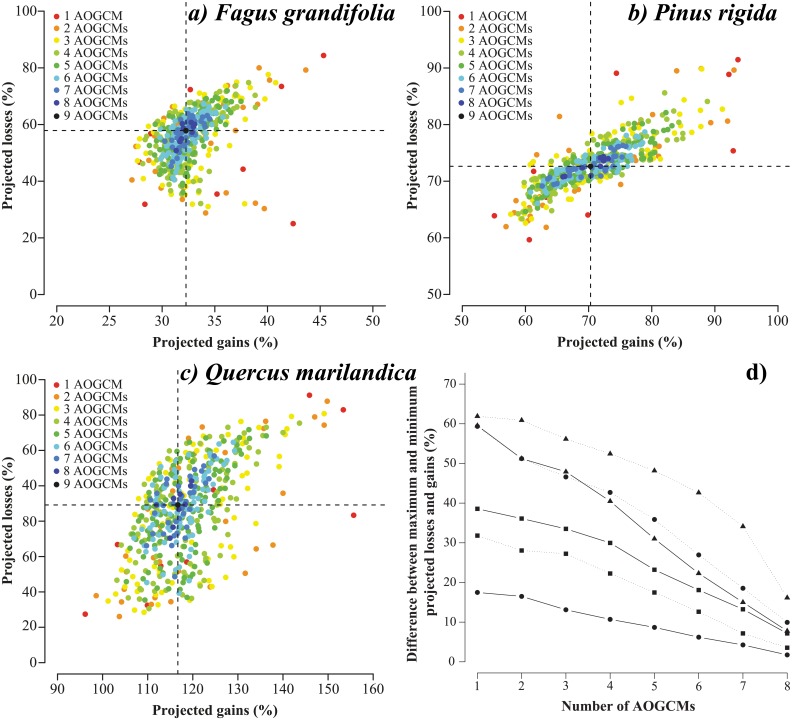
Scatter plots showing uncertainty arising from an arbitrary selection of AOGCMs. Scatter plots (a), (b), and (c) show the projected habitat losses and gains obtained under each ensemble forecasting realized with one to nine AOGCMs for *Fagus grandifolia*, *Pinus rigida*, and *Quercus marilandica*, respectively (dashed lines show average projected losses and gains). Scatter plot (d) represents differences between maximum and minimum projected losses (dashed lines) and between maximum and minimum projected gains (solid lines) for *Fagus grandifolia* (circles), *Pinus rigida* (squares), and *Quercus marilandica* (triangles) using one to eight AOGCMs.

Both percentages of gains and percentages of losses in species habitat distribution were highly variable and depended on the number and choice of AOGCMs (Fig 4). Moreover, even using a high number of AOGCMs, uncertainty in projected species range could be very important. For example, the projected habitat loss of *Quercus marilandica* varied from 40% to 82% of pixels according to the random set of six AOGCMs used to estimate potential future loss (dark green dots in Fig 4c). Negative trends between the number of AOGCMs and the range of changes (maximum minus minimum values) in species habitat distribution (Fig 4d) showed that increasing the number of AOGCMs (that is, better taking into account the uncertainty originating from AOGCMs) reduced uncertainty in the projected change on species habitat distribution.

### Mapping spatial differences

Under a weighted average performed on the six climate change scenarios selected by the k-means algorithm, *Fagus grandifolia* habitat was projected to gain 35.4% of pixels (compared to 32.3% when considering all 27 climate change scenarios) and to lose 48.6% of pixels (compared to 57.9%). Corresponding values were 75.8% versus 70.3% for gains and 70.9% versus 72.6% for losses in the case of *Pinus rigida*, and 121.9% versus 116.7% for gains and 57.0% versus 64.6% for losses in the case of *Quercus marilandica*.

**Fig 5:**
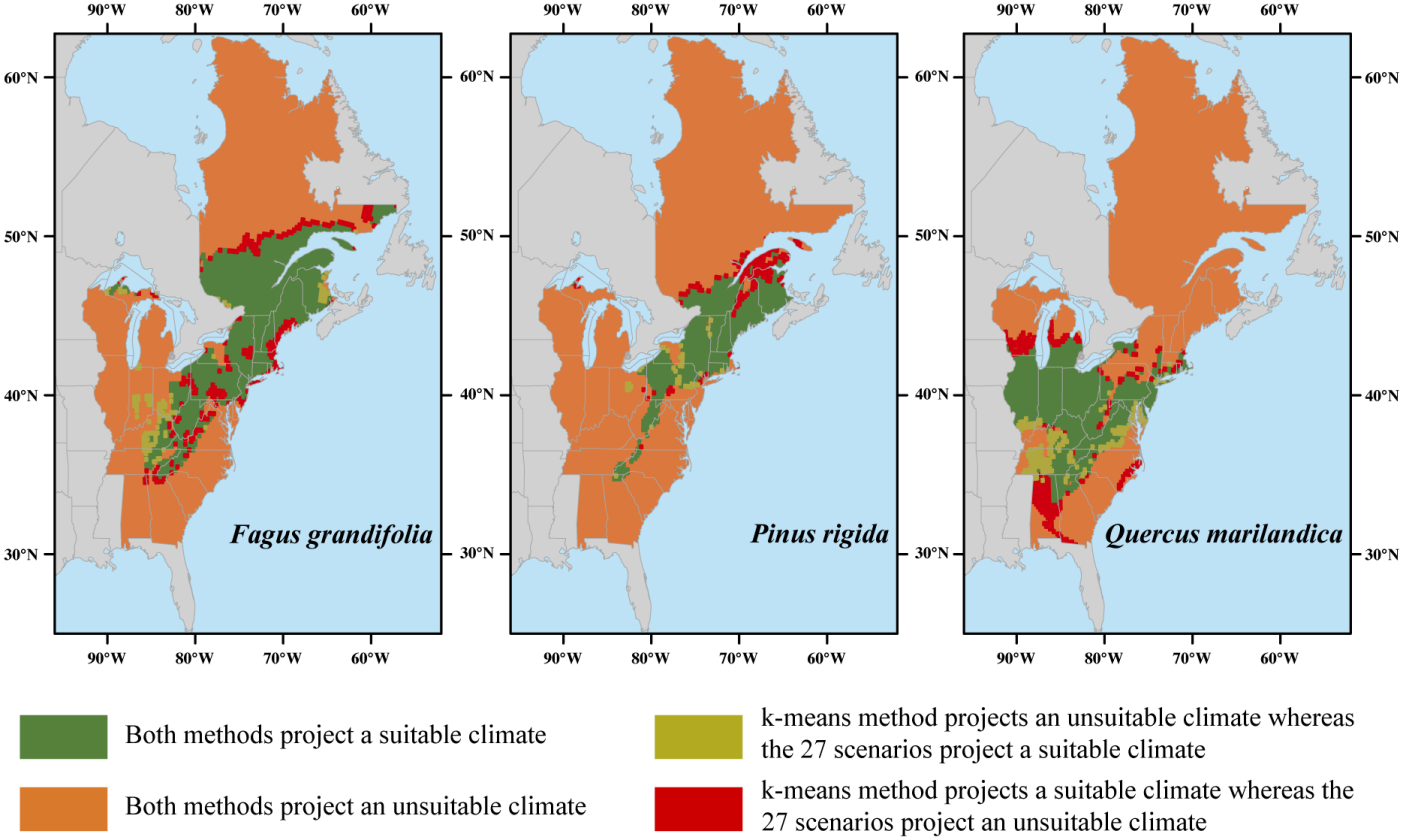
Effects of k-means clustering on potential future species distributions. Maps show differences between the projected climatic habitat distributions (2071–2100) obtained under an ensemble forecasting with the 27 climate change scenarios and an ensemble forecasting with the six climate change scenarios selected by the k-means algorithm for the three tree species.

We compared the potential future habitat distribution projected by the 27 climate change scenarios with the one projected under the six climate change scenarios selected by the k-means approach (Fig 5). This shows that spatial differences are located at the leading and rear edges of the species range, whatever the species considered. More specifically, the weighted average performed on the six climate change scenarios overestimated the projection obtained under a weighted average performed under the 27 climate change scenarios by predicting a more pronounced northward shift.

## Discussion

### Benefits of the k-means clustering approach

Our results show that a reduced number of six climate change scenarios selected by the k-means clustering approach generate average climatic conditions very close to those obtained from of the full set of 27 climate change scenarios available before reduction. In addition, although some discrepancies did appear at the edges of future tree species habitat distributions when comparing projected distributions obtained with the full set of scenarios versus the reduced set (Fig 5), future tree habitat distributions were overall very similar. Our study represents one of the very first applications of the k-means clustering approach in climate change biology. It also provides clear guidance to choose objectively a reduced number of climate change scenarios among the many available alternatives.

The k-means clustering enables a significant reduction of redundancy between the most similar climate change scenarios because it decreases the number of climate change scenarios while retaining the coverage of uncertainty in future climate conditions. This is important because many sources of climate uncertainty exist beyond AOGCMs and forcing scenarios, considered here. For instance, initial conditions of AOGCMs (Table 1) also contribute to future climate uncertainty [42]. Addressing this uncertainty requires multiple runs of the same AOGCM-forcing scenario combination for which initial conditions are slightly perturbed. Another source of uncertainty originates in the downscaling method used to refine AOGCM projections at the regional scale [43]. Statistical downscaling (spatial interpolations after correction for topographic, hydrographic and geographical effects) and dynamic downscaling (regional climate models) can be used, with potential effects on projections of species distribution under climate change assumptions. Therefore, the initial set of climate change scenarios considered by the k-means clustering approach could be increased to include AOGCM, forcing scenario, AOGCM run, and downscaling method as uncertainty factors [18]. It is also noteworthy that although the global mean temperature response simulated by CMIP5 and the preceding CMIP3 (there was no CMIP4) models is very similar, the range of temperature change across all scenarios is wider in AR5 than in AR4 because the RCPs include a strong mitigation scenario (RCP2.6) that had no equivalent among the SRES scenarios. In addition, CMIP5 has more than twice as many models as CMIP3 [44]. This again suggests that our proposed method might gain relevance in the years to come, when attention to alternative climate trajectories might increase among climate change biologists.

Studies using several forcing scenarios usually present future projections with the implicit assumption that each forcing scenario generates a different family of projections [6, 45, 46]. Here we aggregated projections from multiple SDMs, multiple AOGCMs and multiple forcing scenarios, and found that composition of clusters was cutting across families formed by forcing scenarios or AOGCMs (Fig 2). It is thus much more informative for practitioners to see a range of climate change scenarios (and associated projections of e.g. species distributions) that represents the full variability of available climate change projections, rather than a range of climate change scenarios than simply reflects the range of available forcing scenarios.

Garcia *et al.* [19] recently used what they called a “central cluster” approach to summarize the general tendencies among 17 AOGCMs without losing higher order variability reflected in extreme projections. They assessed similarities among AOGCM simulations for each variable projected in the late-century, then grouped co-varying projections before averaging them, and finally used k-means to partition AOGCMs into groups of co-varying projections. Our proposed approach, derived independently, differs in that we selected the existing climate scenarios located closest to each cluster’s center, whereas they projected species distributions from “artificial” climate scenarios that were averages obtained from each cluster. This may be an important difference for some biodiversity managers, who need to communicate projections of species distributions while they are still attached to some existing climate scenarios. Our approach also differs in that we averaged future species habitat distributions while weighing for both performance of statistical models and number of climate scenarios within clusters, while the latter was not a weighing factor in Garcia *et al.* [19]. Biodiversity managers generally prefer to give less importance to the less extreme climate scenarios, although each is equally plausible. Another difference is that the selection of climate scenarios presented in Garcia *et al.* [19] is more spatially explicit than ours. We recognize that this as an avenue for future development of our proposed method, especially at large spatial scale. Indeed, differences in projected climate change are spatially explicit and it would be relevant to take into account this spatial structure to define similar projections. Given the increasing wealth of climate scenarios available for ecological modelling studies, we urge others to build on our efforts and on those of Garcia *et al.* [19].

### Pitfalls of arbitrary selection of climate change scenarios

Projected changes in species habitat distribution were highly variable when climate change scenarios were selected arbitrarily. This was observed even when incorporating several AOGCMs in the process of projecting potential changes in species habitat distribution (Fig 4), although uncertainty in the projected future species habitat distribution was reduced when the analyses included more AOGCMs. Again, this is problematic given that biodiversity managers need robust projections.

Other studies have investigated uncertainty in species distribution projections [47–50] but, to our knowledge, ours is the first exploration of the consequences of an arbitrary selection of AOGCMs on projected species distribution. Our results emphasize both the need to use multiple climate change scenarios to project species distribution in time, and the need to use an appropriate method to select among climate change scenarios. This is particularly true when climate-induced changes are assessed on a large number of species and when a reduced number of climate change scenarios has to be selected.

### Conclusions

The use of a clustering approach to select an objective subset of climate change scenarios offers an appropriate and efficient guidance to project species distribution through time. This method should be most useful to select an appropriate subset of climate change scenarios in the context of regional impact studies, because the realism of climate change scenarios is region-specific and their arbitrary selection could lead to a misrepresentation of future climate possibilities at the regional scale. We also argue that the approach presented here is relevant for a wide range of studies outside the field of climate change biology, such as those dealing with the effects of climate change on transportation infrastructures, human health, or economic systems.

## Supporting Information

S1 Text Description of the “change field” method used to obtain temperature and precipitation data for 2071–2100. (PDF)

S2 Text Description of the method used to select the optimum number of clusters. (PDF)

S3 Text Current ranges predicted for the reference period. (PDF)

S4 Text R script to perform a k-means algorithm initialized with a hierarchical clustering. (TXT)

S5 Text Data of table 2 used with the R script described in S4 Text. (TXT)
