# Supplementary material for: An Objective Approach to Select Climate Scenarios when Projecting Species Distribution under Climate Change
Source: PLoS One. 2016 Mar 25;11(3):e0152495. doi: 10.1371/journal.pone.0152495 (PMC4807766; doi:10.1371/journal.pone.0152495)
Supplement: S1 Text — (PDF) [file pone.0152495.s001.pdf]

**S1 Text – Description of the "change field" method used to obtain temperature and precipitation data for 2071–2100**

This supporting information is linked to the following paper: Casajus, N, Périé, C, Lambert, M-C, de Blois, S and Berteaux, D. An objective approach to select climate scenarios when projecting species distribution under climate change. Submitted to PlosONE (August 2015).

We created future climate scenarios for the temperature and precipitation variables for the period 2071–2100 using the "change field" method [1]. Monthly mean differences (delta values) between the reference period (1961–1990) model run and the future climate model run were calculated and then applied to baseline values of observed monthly climate data for the reference period. However, due to the relatively coarse spatial resolution of the climate simulations ($\approx$ 250 km per cell side), we created interpolated monthly delta values for the 20 km x 20 km grid cell centroids using a linear triangle-based interpolation method based on Delaunay triangulation [2] between climate model grid cell centroids. This function fits a triangulated mesh surface of the form $z = f(x, y)$ to the AOGCM grid cell centroid vectors (x = longitude, y = latitude, z = delta value), and subsequently performs linear interpolation of this surface at the points specified by ($x_i$, $y_i$) to produce $z_i$ (where $x_i$ and $y_i$ represent longitude and latitude values of the 20 km x 20 km grid and $z_i$ are the interpolated delta values). We subsequently applied interpolated delta values to each observed grid cell value to create future climate scenarios for each month.

# References


1. IPCC (2001) Climate Change 2001: Impacts, Adaptation and Vulnerability. Contribution of working group II to the third assessment report of the Intergovernmental Panel on Climate Change. McCarthy JJ, Canziani OF, Leary DJ N A Dokken, White KS, editors. Cambridge, United Kingdom and New York, NY, USA, Cambridge University Press.

2. de Berg M, Cheong O, van Kreveld M, Overmars M (2008) Computational Geometry: Algorithms and Applications. Berlin/Heidelberg, Springer-Verlag.
