# Supplementary material for: An Objective Approach to Select Climate Scenarios when Projecting Species Distribution under Climate Change
Source: PLoS One. 2016 Mar 25;11(3):e0152495. doi: 10.1371/journal.pone.0152495 (PMC4807766; doi:10.1371/journal.pone.0152495)
Supplement: S2 Text — (PDF) [file pone.0152495.s002.pdf]

S2 Text – Description of the method used to select the optimum number of clusters

This supporting information is linked to the following paper: Casajus, N, Périé, C, Lambert, M-C, de Blois, S and Berteaux, D. An objective approach to select climate scenarios when projecting species distribution under climate change. Submitted to PlosONE (August 2015).

Several approaches have been developed to determine how many clusters should be chosen in a k-means clustering. Here we adopt an approach based on a trade-off between costs (i.e. the number of clusters) and benefits (explained variance). Our purpose is to identify the number of clusters from which the net benefit decreases.

The first step is to perform as many k-means as we have objects to classify, by varying the number of selected clusters, from 1 to the total number of objects. For each k-means, we calculate the Rsq statistic as the ratio of the between-group sums of squares to the total sums of squares. This statistic quantifies the amount of variability explained by the clustering.

The second step consists in plotting the Rsq statistic as a function of the number of clusters (Fig. S1). A characteristic of any clustering technique is that this curve describes a concave profile (i.e. a logarithmic curve) bounded between 0 and 1. In this type of profile, the benefits (i.e. the Rsq statistic) increase quickly for a small increase of the costs (i.e. the number of clusters) up to the inflexion point. In other words, the real benefits increase. Beyond this inflexion point, the benefits increase more slowly than the costs, and the real benefits thus decrease. This inflexion point must be found without adjusting any mathematical function.

To do this, we plot the line going through the points (1, 0) and (n, 1), with n the maximum number of clusters (Fig. S1). This line describes a constant increase of benefits, since the benefits and the costs increase with the same increment all along the line. By subtracting the Rsq profile to this line, we obtain the curve of the real benefits (i.e. the net benefits; Fig. S1). The maximum value of this curve corresponds to the inflexion point of the Rsq profile.

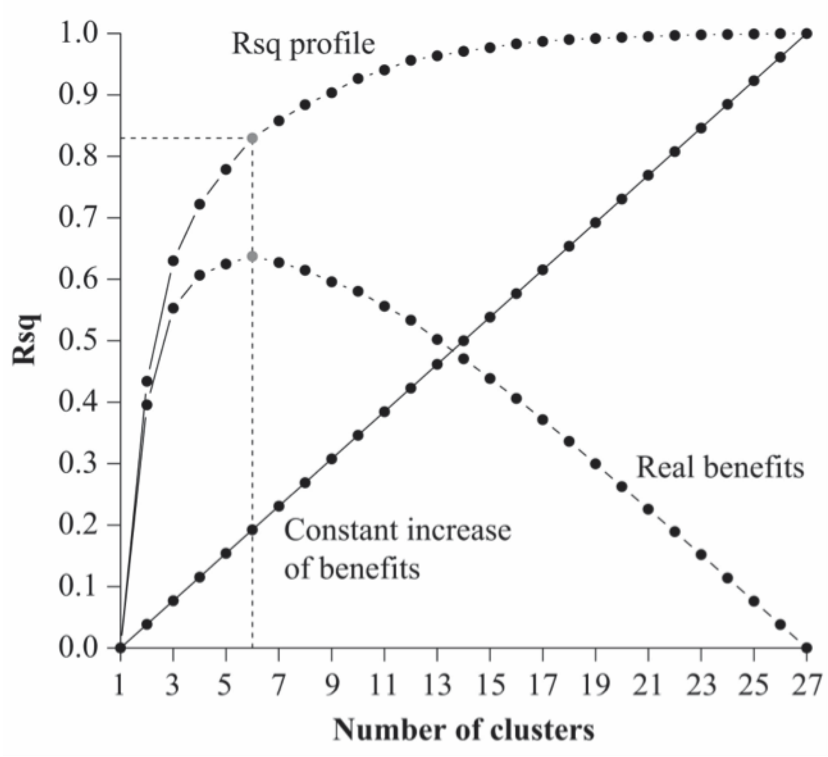

Fig. S1 – Conceptual approach to select the optimum number of clusters

In the example shown in Fig. S1, the optimum k-means clustering led to six clusters explaining 83% of the total variance.
