# Supplementary material for: An Objective Approach to Select Climate Scenarios when Projecting Species Distribution under Climate Change
Source: PLoS One. 2016 Mar 25;11(3):e0152495. doi: 10.1371/journal.pone.0152495 (PMC4807766; doi:10.1371/journal.pone.0152495)
Supplement: S3 Text — (PDF) [file pone.0152495.s003.pdf]

S3 Text – Current ranges predicted for the reference period

This supporting information is linked to the following paper: Casajus, N, Périé, C, Lambert, M-C, de Blois, S and Berteaux, D. An objective approach to select climate scenarios when projecting species distribution under climate change. Submitted to PlosONE (August 2015).

The three following maps show the predicted range of *Fagus grandifolia* (Fig. S2), *Pinus rigida* (Fig. S3) and *Quercus marilandica* (Fig. S4) for the reference period. We indicate good classification in orange (models predict absence when absence is observed) and green (models predict presence when presence is observed) and misclassifications in light green (models predict presence when absence is observed) and red (models predict absence when presence is observed).

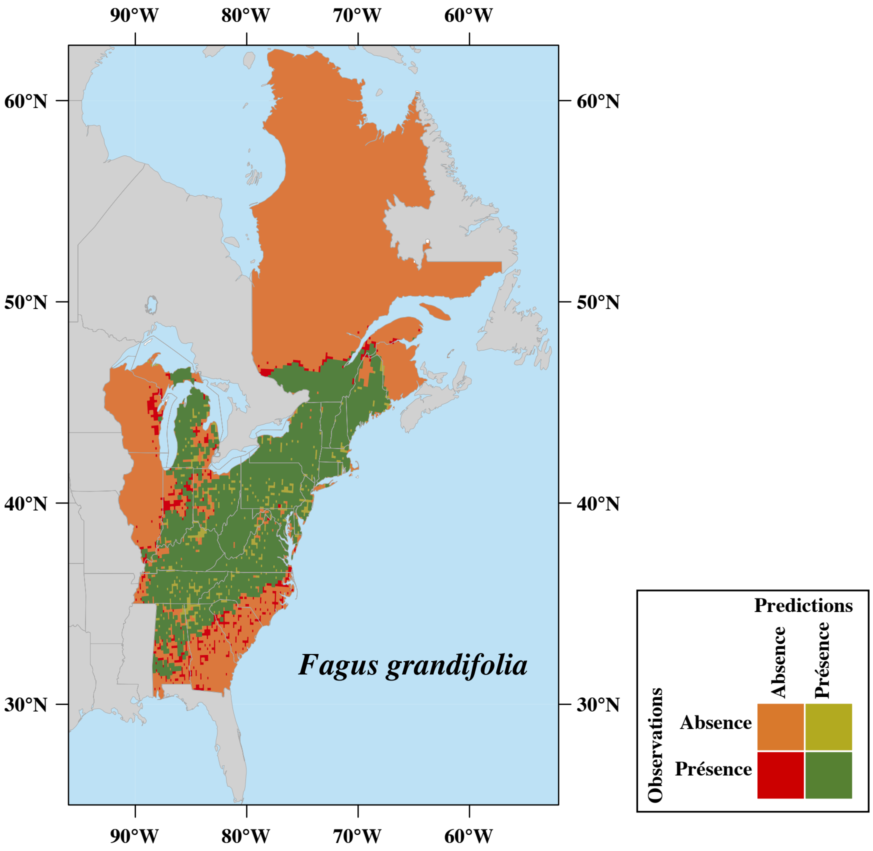

Fig. S2 – *Fagus grandifolia* predicted range for the reference period

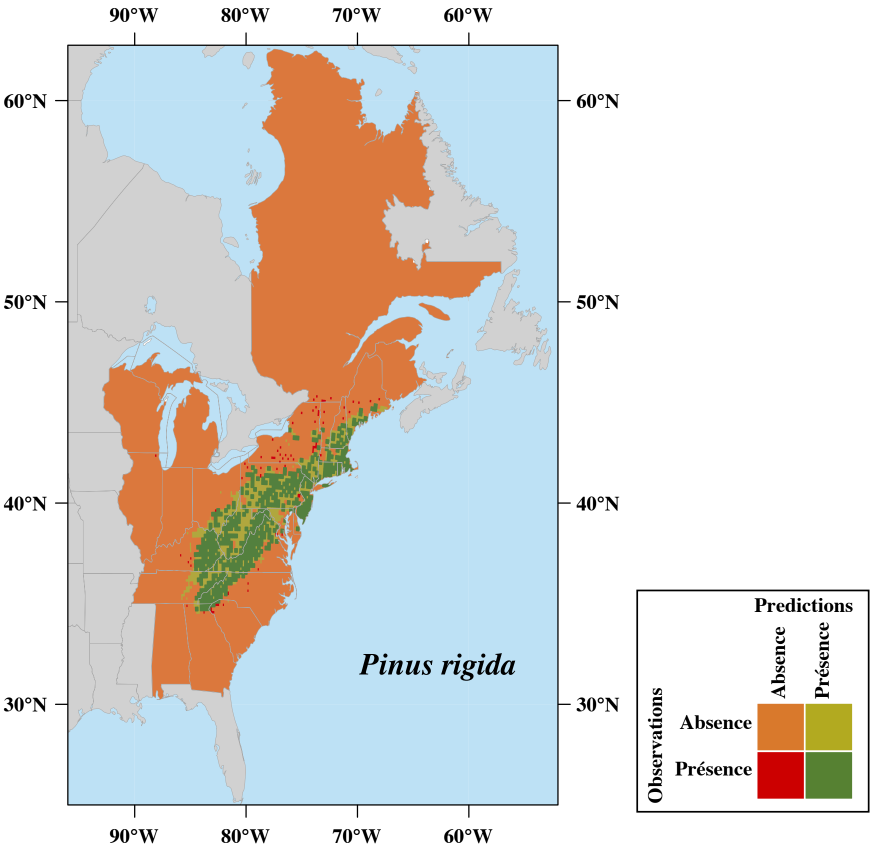

Fig. S3 – *Pinus rigida* predicted range for the reference period

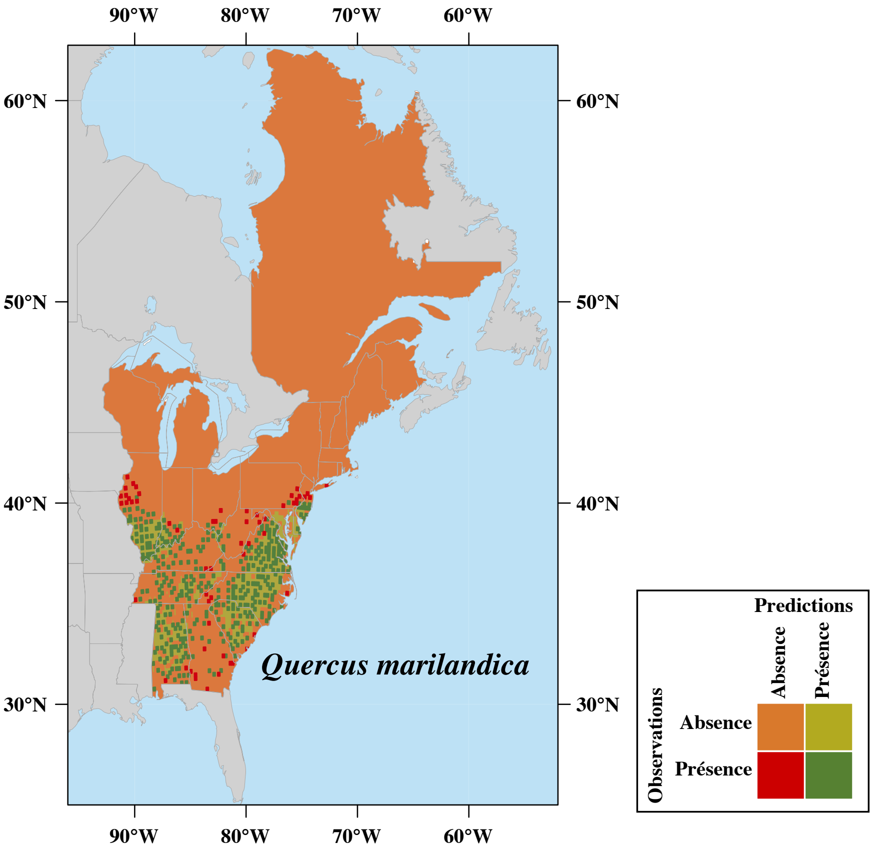

Fig. S4 – *Quercus marilandica* predicted range for the reference period
